# AIN to PIN transfer for PIN palsy following distal biceps tendon repair: a case report

**DOI:** 10.1080/23320885.2022.2096614

**Published:** 2022-07-19

**Authors:** Jillian A. Fairley, Parham Daneshvar

**Affiliations:** a Department of Orthopaedic Surgery, University of British Columbia, Vancouver, British Columbia, Canada; b Department of Orthopaedic Surgery, St Paul's Hospital, Vancouver, British Columbia, Canada

**Keywords:** Nerve transfer, PIN palsy, anterior interosseous nerve, posterior interosseous nerve, distal biceps tendon

## Abstract

Posterior interosseous nerve injury after distal biceps repair significantly impairs hand function. For treatment, we describe an anterior interosseous nerve to posterior interosseous nerve transfer. Our technique is useful when the injury is too distal for median nerve transfer or when the zone of injury is too long for nerve graft reconstruction.

## 1. Introduction

Distal biceps tendon ruptures are commonly repaired surgically using either a one- or two-incision technique. Both approaches are associated with potential complications, including a risk of posterior interosseous nerve (PIN) injury estimated at 1–3% [1–3]. While most PIN injuries are self-resolving neuropraxias, permanent injury can occur [3,4].

Patients sustaining PIN injury can be left with devastating functional loss including loss of finger extension, thumb abduction and extension, and weakened wrist extension with associated radial deviation [1,2,5]. Treatment options for these deficits include tendon transfers, nerve grafts, and nerve transfers, or a combination thereof [3,5–8]. The decision between options depends on patient factors as well as the location, timing, mechanism and extent of injury [8].

**Figure 1.**
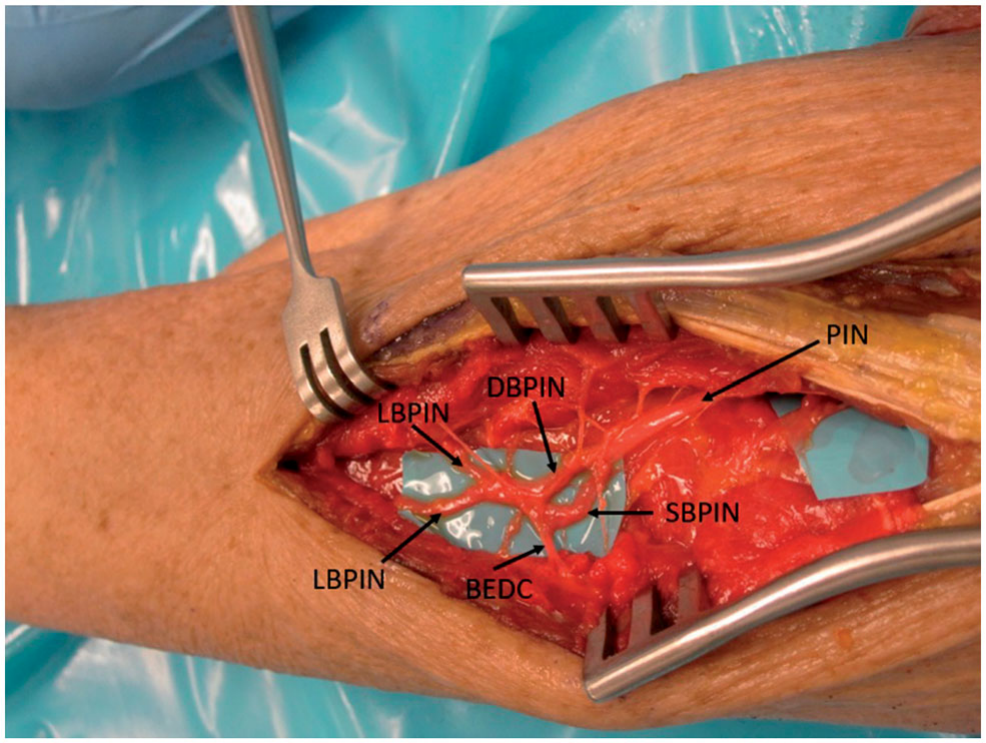
The PIN is followed distally where it branches into the deep (DBPIN) and superficial (SBPIN) branches of PIN. The DBPIN then divides into medial (MBPIN) and lateral (LBPIN) branches. The branch to EDC (BEDC) is also shown.

The PIN branches as it goes deep to the superficial head of the supinator, giving branch(es) to the supinator, and then branching into the deep and superficial heads of the PIN (Figure 1) [9]. According to Bertelli et al. [9] the superficial head gives branches to extensor digitorum communis (EDC), extensor carpi ulnaris (ECU) and extensor digiti minimi (EDM), and the deep head further branches into medial and lateral branches of the PIN. The medial branch innervates extensor indicis proprius (EIP) and extensor pollicis longus (EPL), and the lateral head innervates extensor pollicis brevis (EPB) and abductor pollicis longus (APL).

**Figure 2.**
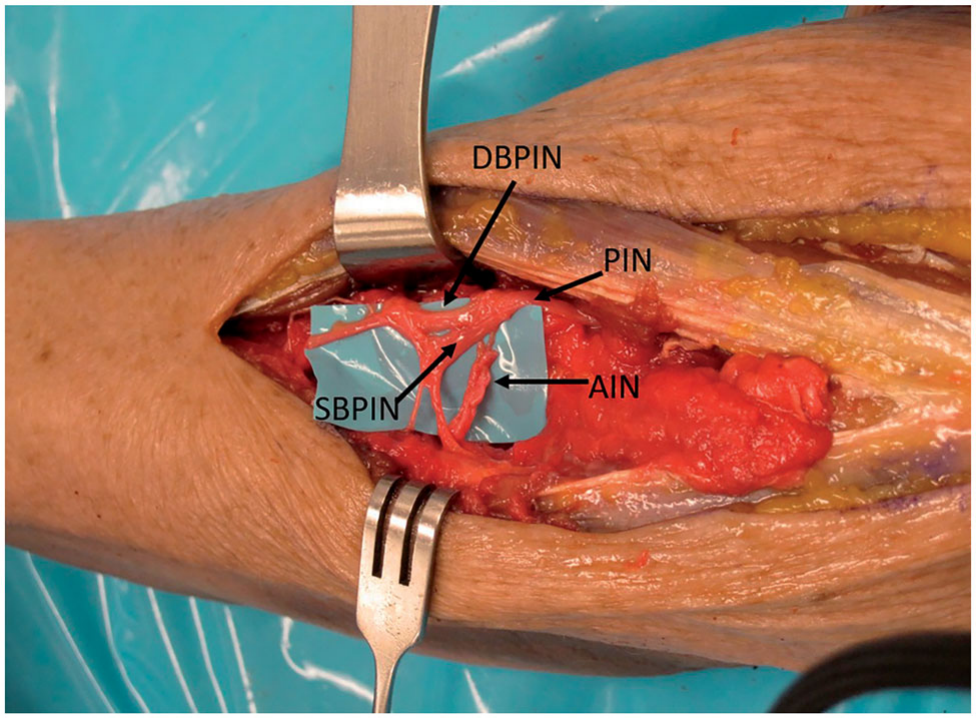
AIN brought dorsally through the interosseous membrane (IOM) where it reaches the PIN just at the level of bifurcation into superficial and deep branches.

Herein, we present the case of a patient who sustained a rare PIN injury after a two-incision distal biceps tendon repair. He subsequently went on to nerve transfer utilizing a novel technique. The donor nerve was the distal extent of the anterior interosseous nerve (AIN) to pronator quadratus (PQ), which is more typically described as a donor option for restoration of intrinsic hand function [10]. However, since sacrifice of PQ leaves little clinical deficit [11], it was a valuable option in this case since its nerve branch provided enough length (Figure 2) to reach the recipient branches of the injured PIN. The distal AIN has been used for transfer in more distal PIN/radial nerve dysfunction, in particular for thumb motion [9]. However, we do not know of any study using the AIN for a more proximal PIN transfer. The transfer more distally is performed to improve thumb function as often there is limited thumb extension recovery with flexorcarpi radialis (FCR) to PIN transfer [9,12]. While FCR is typically used for proximal PIN transfer, this transfer was not performed in our case because the zone of injury was very large and we could only innervate the PIN branches after they had already branched more distally. Thus, the FCR branch was not long enough to allow for the typical median to radial nerve transfer as described by Davidge et al. [13]

## 2. Case presentation

A 46-year-old right-hand dominant male cable technician was referred for evaluation of a PIN palsy. On presentation, he was nearly five months following left distal biceps tendon repair using a two-incision approach. Immediately postoperatively he was unable to extend his fingers and thumb and had radial deviation with wrist extension. These deficits persisted, prompting his original orthopaedic surgeon to order electromyographic (EMG) and nerve conduction studies approximately four months later. These tests demonstrated normal superficial radial sensory signals but absent radial motor signals with no identifiable motor units distal to the supinator.

**Figure 3.**
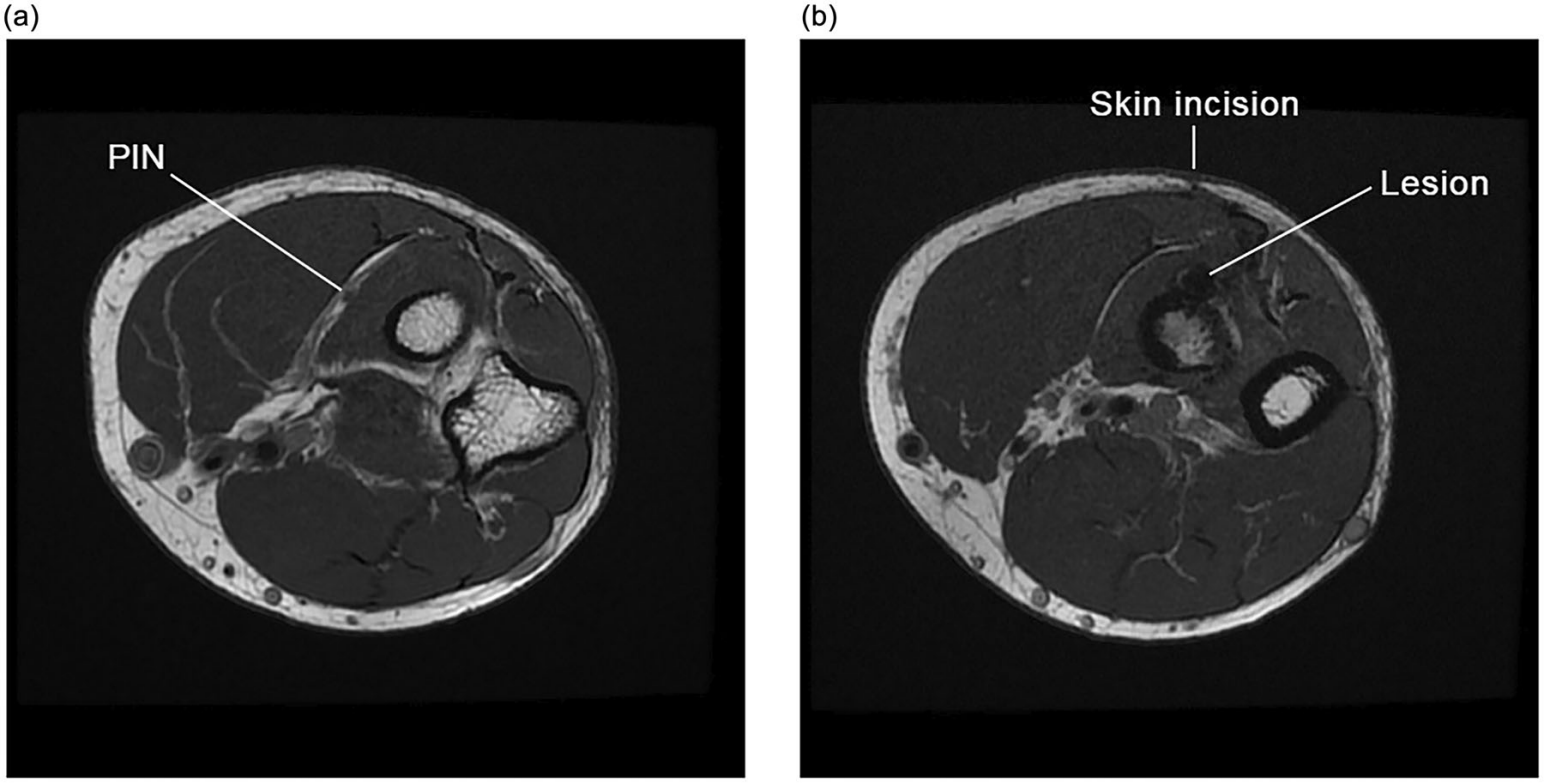
(a,b) Axial T1-weighted magnetic resonance images of the elbow approximately 6 months following a two-incision distal biceps tendon repair demonstrating: (a) an intact more proximal PIN, in its expected location within the fascial plane that will separate the two heads of supinator; and (b) a lesion more distally along the course of the PIN with associated surgical change and suture artifact, along with a more anterior approach than would typically be expected of the posterior incision for a two-incision technique.

The patient was a healthy non-smoker. His initial biceps tendon rupture occurred while loading a heavy toolbox into a truck. Physical examination demonstrated an inability to extend his fingers and thumb, with his wrist extending into radial deviation. Range of motion of his wrist and elbow was maintained except in supination which was limited to 50 degrees of active motion, and 70 degrees of passive motion. His median and ulnar nerves were functioning well. Magnetic resonance imaging (Figure 3(a,b)) was also subsequently performed, which further supported the diagnosis – PIN palsy following two-incision distal biceps tendon repair, with no sign of recovery at 5 months.

After discussing treatment options, the patient chose to proceed with nerve reconstruction, with the option for subsequent tendon transfer if necessary.

## 3. Methods

**Figure 4.**
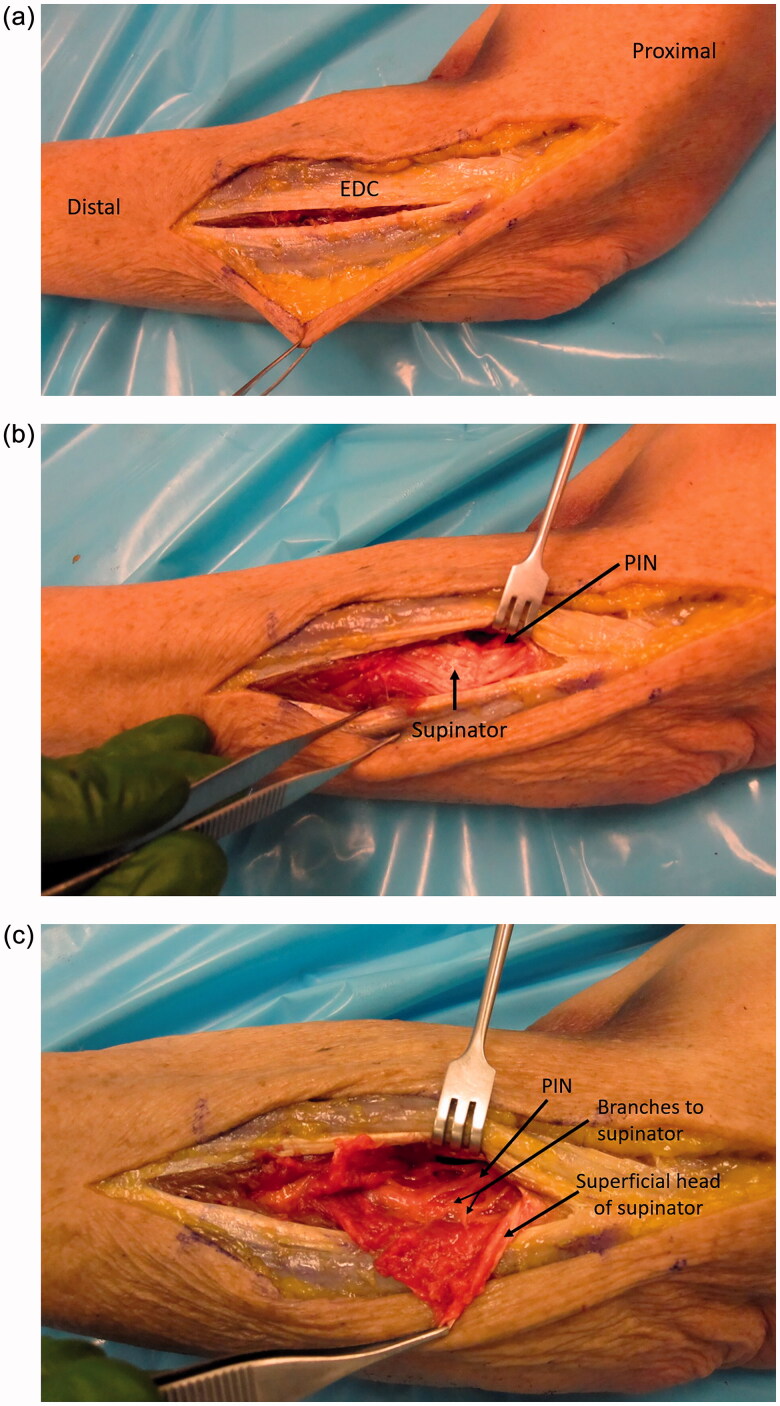
(a) Approach demonstrated in a cadaver specimen. An EDC split approach was used to identify the PIN and zone of injury. This was the location of the approach taken from the patient’s initial distal biceps repair, which was subsequently utilized for the nerve transfer surgery with extension proximally and distally as demonstrated here. (b) Deep to the EDC, the supinator muscle is identified and with the arm in supination, the PIN is brought into view and carefully dissected proximal to the supinator. (c) The superficial head of the supinator is split and the PIN is followed distally.

A lateral incision at the elbow was utilized, extending an existing incision from prior tendon repair proximally to the lateral humeral epicondyle and distally down the forearm. Full-thickness flaps were raised over the extensor mechanism. The extensors were split at the level of the previous surgery (which was a more posterior EDC split), while extending the split proximally and distally (Figure 4(a)). Deep to the extensor mechanism, supinator was identified, and the PIN was found proximally (Figure 4(b)). The radial sensory nerve was seen going deep to brachioradialis. The PIN was then followed distally, and the supinator was split to follow the nerve (Figure 4(c)). About one quarter of the way into the supinator, there was extensive scar tissue and a neuroma. The dissection was continued distally and for a few centimeters there was no identifiable fascicles seen. Near the distal end of the supinator and further distal, multiple branches of the PIN were identified going into the extensor muscles. However, only branches were present and not the PIN as a single bundle. The intact branches were followed further distal, to assess their path, and more proximal toward the zone of injury where they were neurolysed from adhesions. There was no continuity with the proximal extent of the PIN.

Utilizing a nerve stimulator, a branch to supinator which was identified proximally was stimulated with no signs of activation. Testing more proximally, the nerve going to extensor carpi radialis brevis demonstrated activation upon stimulation.

Both nerve ends entering and leaving the zone of injury were then debrided until a healthy fascicular pattern was identified. This resulted in a gap of over five centimeters. The distal intact PIN fascicles were felt to be too distal for a median nerve transfer. With the multiple distal fascicles present and a large gap, we did not feel that grafting would yield good results and were considering tendon transfers. However, other nerve transfer options had been discussed in advance including a distal AIN transfer. The patient understood that this option may or may not yield adequate function but would not compromise future tendon transfers.

**Figure 5.**
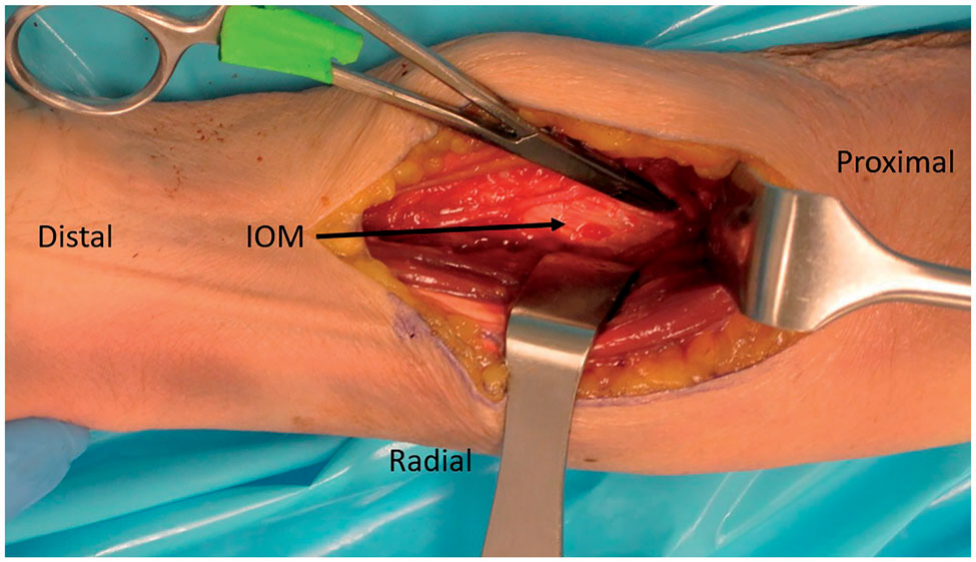
A left cadaveric forearm demonstrating the volar-ulnar exposure. A window through a membranous portion of the IOM, proximal to the central band, is utilized to pass the AIN from anterior to posterior.

**Figure 6.**
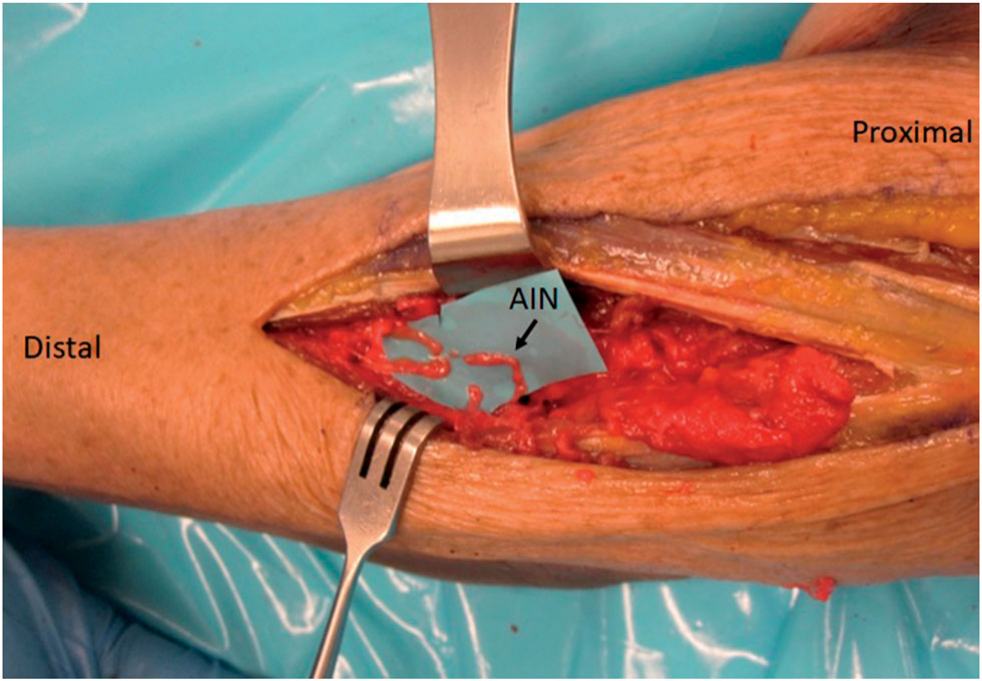
In the case of our patient, three main branches were identified and coapted to the AIN as demonstrated in this representative figure of a left cadaveric forearm.

A second incision was made on the volar-ulnar aspect of the forearm and the distal AIN donor nerve was identified and followed proximally as described for a typical AIN to ulnar motor transfer. We measured the length of AIN and assessed its ability to reach the recipient nerves. Once satisfied that this was feasible, a neurotomy was performed as distal as possible and the AIN was passed from anterior to posterior through a window created in the interosseous membrane just proximal to the central band (Figure 5) which was carefully protected. The window was small, but generous enough to ensure the AIN would not be entrapped by fascia. The nerve was tagged, passed dorsally and coapted tension free to the multiple PIN recipient fascicles (Figure 6) using 9-0 nylon sutures with the assistance of a microscope.

The postoperative course involved the use of a long arm splint for 3 weeks to protect the nerve repair followed by full range of motion. General strengthening started 1 month post surgery. To re-educate for the nerve transfer, the patient was asked to perform finger, thumb and wrist extension while pronating his forearm to activate the AIN branch of the pronator quadratus.

## 4. Results

**Figure 7.**
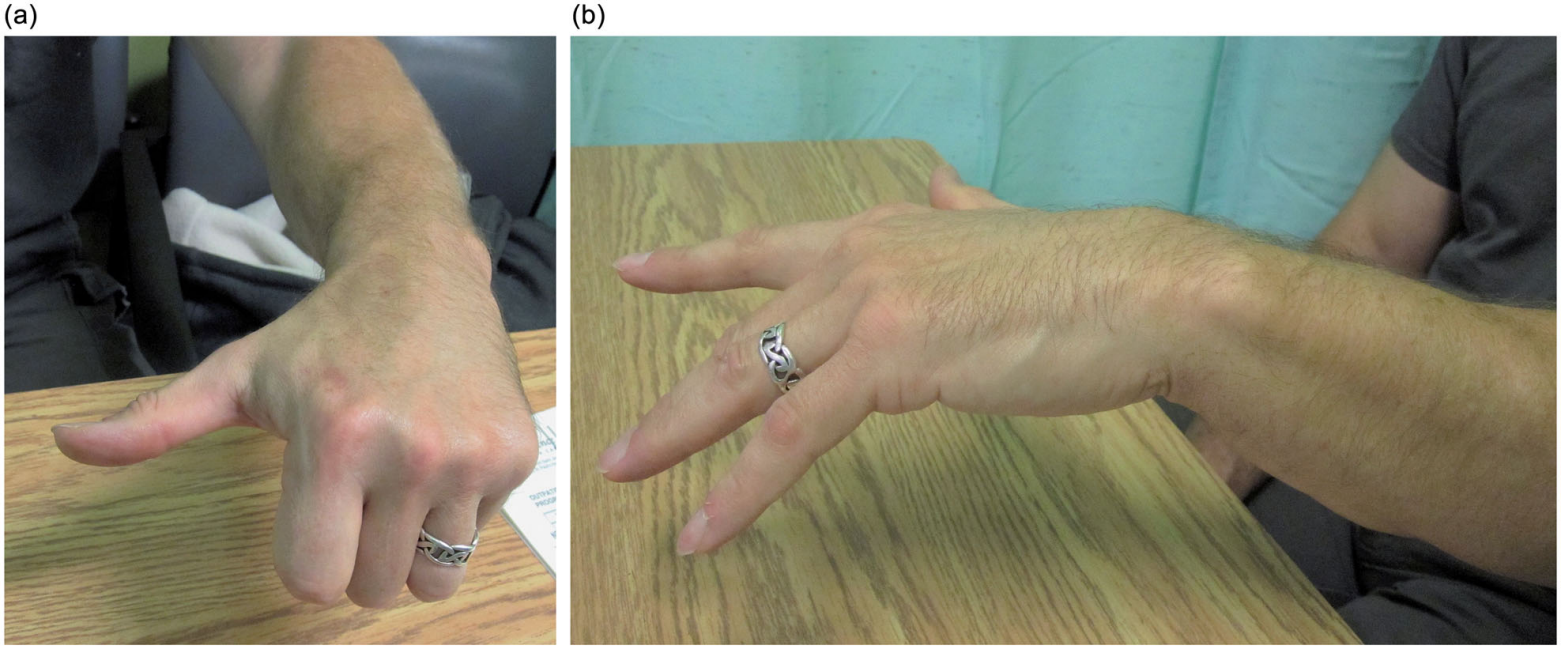
(a) Thumb extension at 1.5 years postoperatively. (b) Finger extension at 1.5 years postoperatively.

At the six-month follow-up from nerve transfer, EMG studies demonstrated the EDC to have some recruitable motor units that increased in number with forearm pronation. At 1.5 years, the patient had good return of thumb and finger extension, with a more central position of wrist extension (Figure 7(a,b); Supplemental Online Video). With a neutral wrist, the thumb had full extension and full strength, the index lacked 20 degrees of extension and had almost full strength, and the remaining fingers lacked 30 degrees of extension and had moderate strength. Although the fingers did not extend all the way against gravity, they had good strength and the patient could open his hand well enough to carry out his manual work.He was pleased with the result and did not feel he needed further surgery, such as a tendon transfers, to improve his function.

## 5. Discussion

Functional deficits from PIN injury are traditionally treated with tendon transfers or nerve grafts when direct repair is not possible [3,5–8]. Mokhtee et al. [5] described a nerve graft reconstruction for two cases of PIN injury following distal biceps tendon repair specifically. They used a medial antebrachial cutaneous nerve graft with good results, restoring thumb and finger extension to both patients with a strength of 4/5 or more. Use of the sural nerve as a graft for PIN injury has also been described [14].

Nerve transfers have gained momentum more recently and, when successful, have demonstrated the ability to return near normal function to the patient. Compared to nerve grafts, transfers have the advantage of only one neurorrhaphy site. They also reduce the distance across which the nerve must regenerate by bringing the donor close to the end target. Although nerve transfers for radial nerve palsy have been described [13], we are unaware of any such reporting on transfer for the entire PIN [9]. The median nerve branches utilized for a typical median to radial nerve transfer may not reach a more distal PIN injury and, in the case of our patient, we did not feel this was an option given how distal the extent of the injury.

A study by Üstün et al. [15] assessed anatomic feasibility of transferring different branches of the median nerve to the PIN. They found the terminal branch to PQ had the ability for neurotization to the PIN. However, our study is the first to report on the implementation and clinical results of this transfer. Ultimately, our patient demonstrated good clinical improvement though full finger extension was not fully restored. We suspect that at least some of this residual loss of finger extension occurred because the zone of injury involved much of the EDC and EDM fascicles and we were unable to find all of the branches from the superficial branch of the PIN (which generally innervate the finger extensors and ECU) for coaptation to our donor AIN. The other reason for incomplete recovery, may have been due to the mismatch in axon counts between donor and recipient nerves as the terminal AIN has about ten times fewer axons than the entire PIN [12,16].

## 6. Conclusion

This case demonstrates the potential of transferring the AIN to the entire PIN as another option for PIN injuries, particularly in the scenario when median nerve transfer or nerve grafting is not feasible.

## Supplementary Material

Supplemental Material
